# CSGL-Former: Cross-Stripes Global–Local Fusion Transformer for Remote Sensing Image Dehazing

**DOI:** 10.3390/s26072102

**Published:** 2026-03-28

**Authors:** Shuyi Feng, Xiran Zhang, Jie Yuan, Youwen Zhu

**Affiliations:** 1 College of Computer Science and Technology, Nanjing University of Aeronautics and Astronautics, Nanjing 210024, China or feng_shu_yi@aliyun.com (S.F.); yuanjie_6952@163.com (J.Y.); 2 Shanghai Spaceflight Institute of TT&C and Telecommunication, Shanghai 201109, China; zhangxiran202602@163.com; 3 Shanghai Aerospace Electronic and Communication Equipment Research Institute, Shanghai 201109, China

**Keywords:** image dehazing, remote sensing, vision transformer, cross-stripes attention, global–local fusion

## Abstract

Remote sensing (RS) images are often degraded by atmospheric haze, which compromises both visual interpretation and downstream applications. To address this, we introduce CSGL-Former, a novel Cross-Stripes Global–Local Fusion Transformer for RS image dehazing. Our model efficiently captures anisotropic long-range dependencies using cross-stripes attention (CSA) and aggregates hierarchical global semantics via a Multi-Layer Global Aggregation (MLGA) module. In the decoder, global context is adaptively blended with fine-grained local features to restore intricate textures. Finally, inspired by the atmospheric scattering model, a soft reconstruction head restores the clear image by predicting spatially varying affine parameters, strictly preserving content fidelity while effectively removing haze. Trained end-to-end, CSGL-Former demonstrates a compelling balance of accuracy and efficiency. Extensive experiments on the RRSHID and SateHaze1K benchmarks show that our model achieves state-of-the-art or highly competitive performance against representative baselines. Ablation studies further validate the effectiveness of each proposed component.

## 1. Introduction

Images acquired under adverse atmospheric conditions, particularly haze, are severely degraded by atmospheric scattering and absorption. The resulting color distortion, contrast suppression, and detail obfuscation not only impede direct visual interpretation but also compromise the reliability of downstream computer vision tasks such as classification, detection, and segmentation. In the domain of remote sensing (RS), where quantitative analysis and geometric fidelity are paramount, haze removal is an indispensable pre-processing step. In real-world RS workflows, image dehazing is rarely an end goal; rather, it is a vital foundation for ensuring the accuracy of subsequent analytics. For instance, in application-driven scenarios such as intelligent transportation systems, adverse atmospheric conditions severely degrade the performance of modern detection frameworks. As comprehensively discussed in recent application-oriented studies, such as the evolution of deep learning-based object detection in RS imagery [1], robust restoration modules are essential to recover critical geometric and semantic features before high-level analysis. Therefore, bridging the gap between low-level image restoration and high-level application accuracy is a primary motivation for our work.

Over the past decades, single-image dehazing has evolved along three main paradigms: image enhancement, physical model-based restoration, and deep learning-based methods. Enhancement approaches, including histogram equalization [2] and Retinex-based formulations [3], improve visibility by manipulating contrast without explicitly modeling the haze formation process. While efficient, they are often brittle under spatially non-uniform haze, leading to artifacts like over- or under-enhancement. Physical model-based methods leverage the atmospheric scattering model (ASM) [4], estimating transmission maps and atmospheric light using statistical priors such as the Dark Channel Prior (DCP) [5] or Color Attenuation Prior (CAP) [6]. However, the underlying assumptions of these priors are frequently violated in RS imagery, which often contains large, bright, or low-texture expanses (e.g., snow, deserts, water bodies), leading to inaccurate parameter estimation and limited generalization.

Recently, deep learning-based methods have achieved state-of-the-art results by learning end-to-end mappings from hazy to clear images. Architectures have progressed from early CNNs (e.g., DehazeNet [7]) to sophisticated designs incorporating multi-scale aggregation and attention mechanisms, and more recently, to powerful Transformer backbones (e.g., DehazeFormer [8]). However, directly applying conventional natural image dehazing architectures to RS imagery presents unique challenges, as the underlying physical assumptions fundamentally differ.

**Figure 1:**
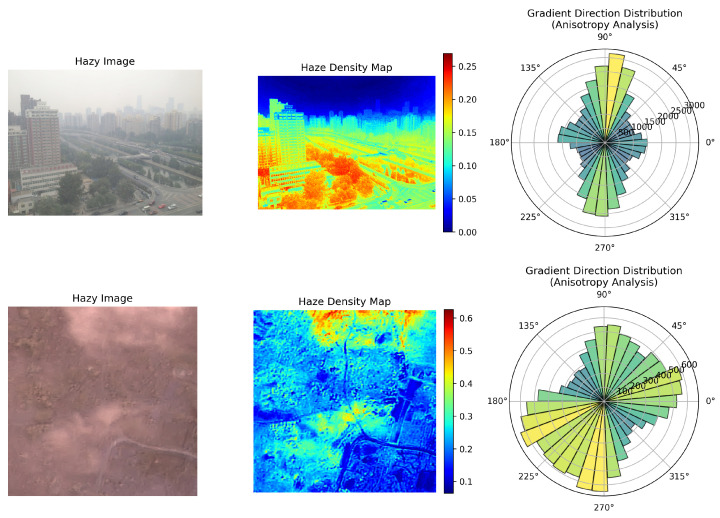
Haze density map and gradient direction distribution for natural (**upper**) and remote sensing (**lower**) images. From left to right: the input hazy image, the haze density map, and the gradient direction distribution. The haze density map is computed as the pixel-wise mean absolute RGB difference between the hazy image and its paired clear reference, serving as an approximation of haze concentration; cooler-to-warmer colors indicate lower-to-higher haze density (see colorbar). The gradient direction distribution is visualized as a polar histogram of high-gradient pixels: angular position denotes gradient direction and radial length denotes occurrence frequency. Bar colors follow frequency for visual emphasis and do not encode an additional variable.

Natural image dehazing typically relies on a depth-dependent haze distribution where density increases continuously with camera depth. In contrast, to address the specific characteristics of aerial scenes, we explicitly formulate two primary domain hypotheses for RS imagery: (i) Hypothesis 1: Anisotropic and Non-homogeneous Haze Distribution. Unlike the horizontal ground-level perspective in natural images, RS images are captured from a top-down (nadir) perspective. Consequently, atmospheric scattering is not dominated by a smooth depth gradient, but rather by spatially non-uniform aerosol concentrations caused by wind patterns, varying terrain elevations, and localized emissions, resulting in a highly anisotropic haze distribution. To empirically validate this assumption, we calculated the haze density and analyzed the gradient direction distribution using polar plots (rose diagrams) for both natural and RS hazy images (as illustrated in Figure 1). In natural images, the rose diagram typically exhibits an extreme single peak at 90° or 270° (the vertical direction), because haze concentration predominantly increases linearly with depth (vertical height). In stark contrast, the rose diagram for RS imagery reveals a broad distribution across all directions (presenting a multi-modal or diffuse pattern). This indicates that the spatial fluctuation of haze in aerial scenes is severe and complex across horizontal, vertical, and even diagonal orientations, definitively confirming its highly anisotropic nature. (ii) Hypothesis 2: Large-Scale Spatial Dependency and Spectral Consistency. RS images typically encompass vast geographical areas containing large, homogeneous regions (e.g., deserts, water bodies, or forests). Conventional CNNs and standard window-based Transformers with limited receptive fields fail to capture the long-range dependencies required to estimate the global atmospheric light consistently across these large expanses, frequently leading to severe color shifts and spectral distortion. Furthermore, the scarcity of paired hazy/clean RS datasets hampers purely data-driven approaches.

To tackle these specific challenges, we propose CSGL-Former, a Cross-Stripes Global–Local Fusion Transformer tailored for RS image dehazing. It synergistically combines cross-stripes attention to model horizontal and vertical haze distributions, Multi-Layer Global Aggregation to accumulate semantic context, and a decoder that meticulously fuses global context from the encoder with local details. A lightweight soft reconstruction head then applies a spatially varying affine transformation to the input image, preserving content integrity while precisely correcting for haze. This holistic design achieves a superior accuracy–efficiency trade-off while maintaining the geometric and spectral consistency crucial for RS applications.

Our main contributions are threefold:

- We introduce a novel cross-stripes global module (CSA + MLGA) that effectively models anisotropic long-range dependencies and aggregates global semantics to handle non-homogeneous haze prevalent in RS images.
- We design an efficient global–local fusion decoder (CGLF + ALEM) that adaptively blends global context from the encoder with local textures from the decoder, enhancing the preservation of fine structures and edges.
- We employ a soft reconstruction strategy that restores the image via a learned affine transformation ($I_{out}\;=\;K⊙I_{in}\;+\;B\;+\;I_{in}$), ensuring content fidelity. Extensive experiments on the RRSHID and SateHaze1K datasets validate the state-of-the-art performance of our method.

## 2. Related Works

### 2.1. Image Enhancement-Based Methods

Enhancement-based dehazing improves visibility without explicitly modeling image formation. Typical techniques include global/local contrast manipulation (histogram equalization [2], CLAHE [9]), Retinex-based illumination-reflectance decomposition [3], and multi-scale or frequency-domain formulations with wavelets [10]. Edge-preserving filters (guided/bilateral) [11] and multi-scale fusion [12] further boost local contrast by aggregating complementary responses from differently enhanced inputs.

These methods are simple, training-free, and efficient—desirable for onboard UAV scenarios. However, because they do not estimate transmission or airlight, they struggle under spatially non-uniform haze, often causing over-/under-enhancement across regions with different haze densities [13]. Typical artifacts include halos near strong edges, color shifts in bright or sky areas, and loss of radiometric faithfulness—issues particularly detrimental to RS applications where spectral consistency matters for downstream tasks (e.g., classification, change detection). Despite these limitations, recent works have continued to refine enhancement techniques for RS imagery. For instance, adaptive Simple Linear Iterative Clustering (SLIC) superpixels have been combined with improved Retinex algorithms to adaptively enhance local contrast while mitigating severe color distortion in complex aerial scenes [14]. Fundamentally, these methods aim at heuristic perceptual visibility improvement rather than physical radiance recovery. Because they arbitrarily manipulate contrast without physical constraints, they carry significant risks of over-enhancement. This yields radiometrically inconsistent outputs that not only compromise visual aesthetics but also severely degrade the reliability of quantitative RS workflows. Consequently, robustness across diverse atmospheric conditions remains limited.

### 2.2. Physical Model-Based Methods

Model-based approaches ground dehazing in the ASM [4] by estimating transmission and atmospheric light using priors or constraints. The Dark Channel Prior (DCP) [5] infers transmission from the presence of near-zero intensities in haze-free patches. While effective in many natural scenes, the assumption breaks in RS imagery with large bright or low-texture expanses (snow, deserts, water), yielding biased transmission and sky over-darkening.

To improve robustness, subsequent works proposed alternative cues: Haze-Lines [15] exploit linear pixel distributions of shared reflectance under varying haze; CAP [6] leverages statistical relations among brightness, saturation, and depth; and boundary/contextual regularization [16] stabilizes transmission with local constraints and spatial smoothness. Non-local formulations [17] incorporate long-range dependencies to better handle large smooth regions and global airlight variations common in wide-area RS scenes.

Despite these advances, physical priors are sensitive to scene content and atmospheric variability (vertical stratification, anisotropic scattering, aerosol types). In UAV settings with strong spatial haze heterogeneity, mis-parameterization leads to residual haze, detail attenuation, or color bias. Moreover, handcrafted priors may compromise spectral fidelity and geometric detail—two properties critical to RS analytics—unless carefully regularized or augmented with auxiliary cues (e.g., NIR/thermal, DEM-based depth, sky segmentation). To better adapt physical models to the vast spatial extent of aerial imagery, recent studies have proposed heterogeneous atmospheric light priors to explicitly modify the classic scattering model for top-down perspectives [18]. Furthermore, researchers have actively integrated these physical assumptions into deep learning frameworks, utilizing dark channel constraints to guide network optimization and enhance physical interpretability [19].

### 2.3. Deep Learning-Based Methods

Deep learning has substantially advanced RS dehazing by overcoming the limitations of handcrafted priors. Early CNNs like DehazeNet [7] and MSCNN [20] pioneered the estimation of physical parameters. AOD-Net [21] reformulated the ASM into a unified end-to-end framework, while subsequent designs emphasized multi-scale feature propagation and attention mechanisms. For instance, FFANet [22] and GridDehazeNet [23] focused on combining global context with local details, and MSBDN [24] improved feature flow with a boosted decoder design.

The advent of Vision Transformers has pushed performance boundaries further. DehazeFormer [8] leveraged shifted-window self-attention to model long-range dependencies. More relevant to RS, PCSformer [25] introduced cross-stripe attention to efficiently capture anisotropic haze distributions, while Trinity-Net [26] integrated a Swin Transformer with physics-informed priors for joint dehazing and downstream task optimization. Building on these self-attention mechanisms, recent Transformer-based models like LCEFormer explicitly enrich multi-scale local context to complement global self-attention [27]. Concurrently, Generative Adversarial Networks (GANs) have been augmented with specialized color space and texture enhancement modules to better recover radiometric fidelity [28]. Most recently, state space models (e.g., SC-DehazeMamba) have been introduced to the RS dehazing domain, leveraging scale-aware aggregation and direction-aware scanning to efficiently capture hierarchical features with linear complexity [29].

Parallel efforts have focused on mitigating data scarcity in RS dehazing. Hybrid and weakly supervised strategies are common, where models leverage unlabeled real-world data to bridge the synthetic-to-real domain gap. For example, SFSNiD [30] employs a pseudo-label retraining scheme and a novel brightness loss to suppress haze and glow artifacts while preserving realistic illumination. Our work builds upon these advances but focuses on creating a highly optimized architecture that explicitly balances global context aggregation and local detail fidelity for the specific demands of RS imagery. Crucially, while many modern deep learning approaches implicitly behave as perceptual enhancement methods that directly synthesize pixels (acting as black-box enhancers), our proposed CSGL-Former fundamentally aims at data-driven physical radiance recovery. By strictly avoiding aggressive, unconstrained pixel hallucination, our model preserves the radiometric consistency essential for RS analysis.

## 3. Proposed Method

### 3.1. Overall Architecture

**Figure 2:**
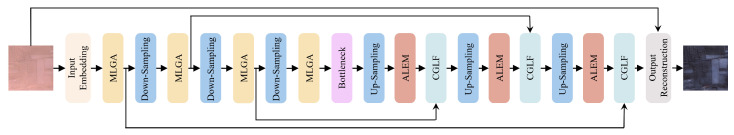
Overall architecture of CSGL-Former.

To address the challenges of non-homogeneous haze, large-scale object context, and local detail preservation in remote sensing imagery, we propose the Cross-Stripes Global–Local Fusion Transformer (CSGL-Former). The network adopts a compact U-Net style encoder–decoder architecture, featuring our proposed cross-stripes global module and adaptive local enhancement. It reconstructs the clear image via a spatially varying affine transformation applied to the input, ensuring content fidelity. The overall framework, depicted in Figure 2, consists of a shallow feature embedding, a 4-stage hierarchical encoder, a bottleneck layer, a 3-stage decoder, and a final soft reconstruction head.

- Input Embedding: A 7×7 convolution with a stride of 2 maps the input image $I_{in}\in R^{H\times W\times 3}$ to a shallow feature map $X_{0}\in R^{(H/2)\times (W/2)\times C}$ (where C=48 by default), providing a strong local inductive bias from the outset.
- Encoder: Four hierarchical stages operate at progressively smaller resolutions $\left\{\frac{1}{2},\frac{1}{4},\frac{1}{8},\frac{1}{16}\right\}$. Each stage comprises a Multi-Layer Global Aggregation (MLGA) block, which contains three stacked CSA layers with learned inter-layer weighting. Between stages, a 2×2 strided convolution performs downsampling while doubling the channel dimension (48→96→192→384).
- Bottleneck: A single cross-stripes attention (CSA) layer followed by a 1×1 convolution and a ReLU activation function refines the highest-level features at the smallest spatial resolution.
- Decoder: Three upsampling stages progressively restore spatial resolution. Each stage uses PixelShuffle with a factor of 2 to double the resolution while halving the channel count (384→96→24→6). Each stage also incorporates an Adaptive Local Enhancement Module (ALEM) and fuses its output with the corresponding encoder feature map via our Cross-Channel Global–Local Fusion (CGLF) module.
- Output Reconstruction: A final 1×1 projection layer maps the decoder output to a 4-channel feature map, which is bilinearly upsampled to the original input resolution (H,W). This map is then split into a modulation map $K\in R^{H\times W\times 1}$ and a residual map $B\in R^{H\times W\times 3}$. The final haze-free image is produced by the soft reconstruction formula $I_{out}=K⊙I_{in}+B+I_{in}$, enabling spatially adaptive correction while preserving the original image content.

Conceptually, our architecture is a theoretically grounded solution designed to address the specific physical characteristics of RS haze. To provide a deeper interpretation, our global–local fusion paradigm explicitly mirrors the inversion process of the Atmospheric Scattering Model (ASM) through three distinct mechanisms:

- The Global Stream (Encoder via CSGM): Haze in aerial scenes is typically non-homogeneous and spatially anisotropic. The encoder acts as a low-frequency estimator to model the large-scale atmospheric veil (i.e., airlight and transmission map variations). By utilizing cross-stripes attention, it efficiently captures the anisotropic distribution of aerosols over vast geographical expanses without being distracted by local surface noise.
- The Local Stream (Decoder via ALEM): While the encoder excels at capturing global context, its large receptive field can inadvertently blur fine-grained local details. To counteract this, the decoder incorporates the Adaptive Local Enhancement Module (ALEM) to act as a high-frequency restorer, strictly preserving the intricate radiometric and geometric textures inherent to RS imagery (e.g., buildings, road networks).
- The Synthesis (CGLF): Our Cross-Channel Global–Local Fusion module acts as an intelligent unmixing mechanism. Rather than performing a naive concatenation, it adaptively modulates the scene radiance (local textures) using the estimated atmospheric distribution (global context), theoretically mirroring the ASM inversion.

Beyond quantitative performance gains, this design offers two distinct conceptual advantages over recent methods: (1) Content Fidelity: By learning a spatially varying affine transformation ($I_{out}=K⊙I_{in}+B+I_{in}$) rather than generating pixels from scratch, we mathematically restrict the solution space to strictly preserve critical topological structures. (2) Interpretability: The explicit separation of global anisotropic atmospheric modeling (CSA) and local feature extraction (DWConv) yields a clearer, more interpretable feature hierarchy than monolithic networks.

### 3.2. Module Designs

#### 3.2.1. Cross-Stripes Global Module (CSA + MLGA)

Standard window-based attention, as used in DehazeFormer, struggles to model long-range dependencies essential for large objects in RS imagery. To provide a clearer differentiation from existing RS-oriented Transformer models, we specifically address the limitations of recent architectures. Conversely, while the cross-stripes attention in PCSformer efficiently captures horizontal and vertical context to handle non-homogeneous haze, it lacks a mechanism for hierarchical global aggregation. Our Cross-Stripes Global Module (CSGM) explicitly addresses this limitation by synergistically combining these ideas. Instead of relying on single-layer attention, we use cross-stripes attention to expand receptive fields along cardinal axes and a Multi-Layer Global Aggregation (MLGA) block to accumulate global semantics across multiple CSA layers. This prevents information redundancy and ensures feature consistency across large objects. This design effectively addresses both large-object modeling and non-homogeneous haze adaptation. The CSGM is composed of a cross-stripes attention (CSA) layer and a Multi-Layer Global Aggregation (MLGA) block.

**I. Cross-Stripes Attention (CSA)**. To efficiently capture long-range dependencies aligned with the typical horizontal and vertical distributions of haze and geographic features in aerial scenes, we adopt cross-stripes attention (CSA). Before partitioning, the input feature is normalized using Layer Normalization. To process arbitrary input resolutions H×W, we employ a dynamic zero-padding strategy. If *H* or *W* is not strictly divisible by the stripe size *s* (default s=4), the feature map is padded at the bottom and right boundaries by pad_h=(s−(Hmods))mods and pad_w=(s−(Wmods))mods.

Horizontal Stripe Attention: The normalized feature map $X_{norm}$ is partitioned into non-overlapping horizontal stripes $\left\{X_{i}^{h}\right\}$ of size $s_{h}\times W$. For each stripe, queries, keys, and values are computed and attention is applied:

(1) $$\left(Q_{i}^{h,k},K_{i}^{h,k},V_{i}^{h,k}\right)=X_{i}^{h}W_{k}^{Q},X_{i}^{h}W_{k}^{K},X_{i}^{h}W_{k}^{V}\;Y_{i}^{h,k}=\mathrm{Softmax}\left(Q_{i}^{h,k}{\left(K_{i}^{h,k}\right)}^{T}/\sqrt{d}\right)V_{i}^{h,k}$$

The outputs from all heads are concatenated and projected, and the resulting features from all stripes are reassembled to form the horizontal attention output $Y^{h}\in R^{H\times W\times C/2}$.

Vertical Stripe Attention: A symmetric process is applied to vertical stripes of size $H\times s_{w}$, yielding the vertical attention output $Y^{v}\in R^{H\times W\times C/2}$.

Stripe Fusion: The horizontal and vertical outputs are concatenated along the channel dimension, fused with a 1×1 convolution, and regularized with a Dropout layer. An internal residual connection is added to produce the final output:

(2) $$\mathrm{CSA}\left(X\right)={\mathrm{Conv}}_{1\times 1}\left(\mathrm{Concat}\left(Y^{h},Y^{v}\right)\right)$$

To mitigate the “inter-stripe isolation” problem, we incorporate a cyclic shift mechanism similar to PCSformer before forming the stripes, ensuring information flow between adjacent stripes in successive blocks.

**II. Multi-Layer Global Aggregation (MLGA)**. Inspired by UAVD-Net, which demonstrates the power of feature aggregation, our MLGA block accumulates global context across three consecutive CSA layers to enhance semantic consistency. Crucially, this dynamic aggregation mechanism fundamentally differs from the generic feature fusion or standard sequential residual connections widely adopted in existing models like DehazeFormer. In natural image dehazing, feature scaling and sequential residuals are primarily employed to stabilize gradient flow. In contrast, RS images suffer from highly non-homogeneous and spatially variant haze, where different regions require varying semantic receptive fields. Our MLGA block is explicitly designed as a dynamic, data-dependent fusion strategy tailored for this physical reality.

Given an input *X*, the process is as follows: Let $X_{1}=\mathrm{CSA}\left(X\right)$, $X_{2}=\mathrm{CSA}\left(X_{1}\right)$, and $X_{3}=\mathrm{CSA}\left(X_{2}\right)$. By embedding the residual connections internally within each CSA layer, we streamline the block’s information flow. We then use channel-wise attention to dynamically weight the output of each layer, preventing information redundancy. Unlike static learned parameters, these fusion coefficients are dynamically generated and conditioned on the specific haze distribution of the input image. This allows the network to adaptively emphasize deeper semantic features for thick haze regions and shallower features for clear regions.

The weights $\left(\alpha_{1},\alpha_{2},\alpha_{3}\right)$ are derived from the sum of the feature maps:

(3) $$\left\{\alpha_{1},\alpha_{2},\alpha_{3}\right\}=\mathrm{Softmax}\left(\mathrm{MLP}\left(\mathrm{GAP}\left(X_{1}+X_{2}+X_{3}\right)\right)\right)$$

where GAP is global average pooling and MLP is a two-layer perceptron. The final aggregated global feature is a weighted sum:

(4) $$\mathrm{MLGA}\left(X\right)=\alpha_{1}X_{1}+\alpha_{2}X_{2}+\alpha_{3}X_{3}$$

#### 3.2.2. Adaptive Local Enhancement Module (ALEM)

While the encoder excels at capturing global context, its large receptive field can inadvertently blur fine-grained local details such as edges and small objects. To counteract this, the Adaptive Local Enhancement Module (ALEM) is introduced in the decoder. ALEM combines lightweight convolutions with an efficient channel-wise attention mechanism and a learnable gate, allowing it to adaptively enhance textures based on the local haze severity.

**I. Local Feature Extraction (LFE).** A stack of three depthwise separable convolutions (DWConv) efficiently extracts low-level local features with minimal computational overhead:

(5) $$F_{lfe}={\mathrm{DWConv}}_{3\times 3}\left(\mathrm{ReLU}\left({\mathrm{DWConv}}_{3\times 3}\left(\mathrm{ReLU}\left({\mathrm{DWConv}}_{3\times 3}\left(X\right)\right)\right)\right)\right)$$

The 3×3 kernel of the DWConv focuses on the 8-neighbor local region, making it highly effective for detail recovery compared to the token-based operations in Transformers.

**II. Adaptive Enhancement (AE).** To incorporate broader spatial context efficiently without the quadratic complexity $O({\left(HW\right)}^{2})$ of standard spatial attention, we introduce a Channel Multi-Dconv Transposed Attention (ChannelMDTA) mechanism. Given the input $X\in R^{H\times W\times C}$, we first apply Layer Normalization to stabilize the feature distribution. Instead of standard multi-head self-attention (MHSA) with explicit positional encoding, ChannelMDTA generates the queries (*Q*), keys (*K*), and values (*V*) using a 1×1 point-wise convolution followed by a 3×3 depthwise convolution:

(6) $$Q,K,V={\mathrm{DWConv}}_{3\times 3}\left({\mathrm{Conv}}_{1\times 1}\left(\mathrm{LayerNorm}\left(X\right)\right)\right)$$

The 3×3 depthwise convolution acts as an implicit positional encoding, effectively capturing local spatial context. To compute attention across the channel dimension, we reshape Q,K, and *V* to dimensions $R^{M\times C_{h}\times N}$, where *M* is the number of heads, $C_{h}=C/M$ is the channels per head, and N=H×W is the spatial resolution. We then apply $L_{2}$ normalization to *Q* and *K* along the spatial dimension. The cross-covariance attention map is calculated as:

(7) $$F_{att}={\mathrm{Conv}}_{1\times 1}\left(\mathrm{Softmax}\left(\tau \cdot (\hat{Q}{\hat{K}}^{T})\right)V\right)$$

where $\hat{Q}$ and $\hat{K}$ are the $L_{2}$-normalized queries and keys, and $\tau \in R^{M\times 1\times 1}$ is a learnable temperature parameter that dynamically controls the sharpness of the attention distribution. By calculating the attention matrix over the channel dimension ($C_{h}\times C_{h}$) rather than the spatial dimension (N×N), ChannelMDTA reduces the computational complexity to $O(HWC^{2})$, making it highly efficient for high-resolution remote sensing images.

A 1×1 convolution then generates a spatially-aware gating map $g\in {\left[0,1\right]}^{H\times W}$ that adaptively interpolates between the convolutional feature ($F_{lfe}$) and the attentional feature ($F_{att}$):

(8) $$g=\mathrm{Sigmoid}\left({\mathrm{Conv}}_{1\times 1}\left(X\right)\right)\;F_{ae}=g⊙F_{lfe}+\left(1-g\right)⊙F_{att}$$

This gating mechanism allows the module to dynamically prioritize local, high-frequency details from LFE or global contextual information from ChannelMDTA depending on the scene content. The final enhanced local feature is produced with a residual connection:

(9) $$\mathrm{ALEM}\left(X\right)={\mathrm{Conv}}_{1\times 1}\left(F_{ae}\right)+X$$

#### 3.2.3. Cross-Channel Global–Local Fusion (CGLF) Module

A critical aspect of U-Net architectures is the fusion of encoder and decoder features via skip connections. Encoder features provide rich global context and haze distribution cues, while decoder features emphasize fine-grained local details necessary for high-quality reconstruction. Our approach to this global–local modeling fundamentally differs from recent RS-oriented architectures like Trinity-Net. While Trinity-Net relies on a Swin Transformer backbone integrated with handcrafted, physics-informed priors, we avoid such dependencies. Instead of naive concatenation or relying on explicit, potentially brittle physical priors, our Cross-Channel Global–Local Fusion (CGLF) module intelligently and adaptively merges these two streams. It computes soft, data-dependent weights for each branch and preserves a residual path for the local feature stream to ensure robust detail fidelity.

Given an encoder feature map $F_{g}$ and a decoder feature map $F_{l}$ at the same spatial resolution, we first align their channel dimensions using separate 1×1 convolutions:

(10) $$F_{g}^{\prime }={\mathrm{Conv}}_{1\times 1}\left(F_{g}\right),\;F_{l}^{\prime }={\mathrm{Conv}}_{1\times 1}\left(F_{l}\right).$$

Next, we create a mixed representation $F_{mix}=F_{g}^{\prime }+F_{l}^{\prime }$ and apply global average pooling followed by a 2-layer MLP to generate two softmax-normalized scalar weights, $\left(\omega_{g},\omega_{l}\right)$, for the entire sample. The final fusion is performed as:

(11) $$\mathrm{CGLF}\left(F_{g},F_{l}\right)=\omega_{g}F_{g}^{\prime }+\omega_{l}F_{l}^{\prime }+F_{l}^{\prime }.$$

The additional $F_{l}^{\prime }$ term acts as a residual connection, ensuring that the decoder’s high-frequency local details are always preserved and form the backbone of the fused representation.

#### 3.2.4. Soft Reconstruction Module

Inspired by recent advances in image restoration, we adopt a soft reconstruction mechanism instead of directly predicting the final RGB image. This approach reconstructs the clear image by predicting spatially varying affine transformation parameters and applying them to the original hazy input. Unlike traditional enhancement methods (e.g., Retinex or histogram equalization) that heuristically manipulate contrast and often cause color shifts or artifacts under non-uniform haze, our method explicitly falls under the paradigm of data-driven physical restoration approximation. This preserves the high-frequency content of the input image and avoids the blurriness often associated with direct pixel synthesis.

Specifically, a final 1×1 convolution maps the last decoder feature map to four channels. This map is then bilinearly upsampled to the original input resolution (H,W) and split into a multiplicative modulation map $K\in R^{H\times W\times 1}$ and an additive residual map $B\in R^{H\times W\times 3}$. The final haze-free output is computed as:

(12) $$I_{out}=K⊙I_{in}+B+I_{in},$$

where ⊙ denotes element-wise multiplication. To theoretically articulate the connection between this spatially adaptive affine correction and the classic Atmospheric Scattering Model (ASM), we recall the standard ASM formulation:

(13) $$I_{in}=J⊙t+A\left(1-t\right)$$

where *J* is the clear image, *t* is the transmission map, and *A* is the atmospheric light. By algebraically rearranging this equation to solve for the clear image *J*, we obtain:

(14) $$J=I_{in}⊙\frac{1}{t}-A\frac{1-t}{t}$$

Our soft reconstruction formula can be equivalently rewritten as $I_{out}=\left(K+1\right)⊙I_{in}+B$. By comparing the two equations, the physical correspondence becomes evident: the modulation term (K+1) acts as a dynamic approximation of the inverse transmission map (1/t), effectively restoring local contrast. Simultaneously, the additive term *B* approximates the atmospheric veil correction −A(1−t)/t. Furthermore, the explicit inclusion of $+I_{in}$ acts as an identity skip connection. In haze-free regions where t≈1, the network simply needs to learn K→0 and B→0, which makes the optimization landscape significantly smoother. By mathematically mirroring the inverse of the atmospheric scattering model, this formulation restricts the network to learn correction parameters rather than synthesizing pixels from scratch. This ensures that the radiometric and structural integrity (content fidelity) of the original RS image is strictly preserved.

### 3.3. Loss Function

While the $L_{1}$ loss provides stable optimization and correlates well with PSNR, relying solely on it often leads to the well-known “regression-to-the-mean” problem, which tends to smooth-out high-frequency spatial details and results in blurry textures. To effectively preserve fine-grained geometric details and enhance perceptual quality, we optimize our CSGL-Former using a combination of the $L_{1}$ loss and a perceptual loss. First, the pixel-wise $L_{1}$ loss between the dehazed output $I_{out}$ and the ground-truth clear image *J* is defined as:

(15) $$L=L_{1}=\frac{1}{HW}\sum_{x,y}|I_{out}\left(x,y\right)-J\left(x,y\right)|.$$

To further recover realistic textures and mitigate over-smoothing, we incorporate a perceptual loss $L_{per}$. It computes the $L_{1}$ distance between the deep feature maps of $I_{out}$ and *J* extracted by a pre-trained VGG-19 network:

(16) $$L_{per}=\sum_{i}\frac{1}{C_{i}H_{i}W_{i}}|\phi_{i}\left(I_{out}\right)-\phi_{i}\left(J\right)|$$

where $\phi_{i}\left(\cdot \right)$ denotes the feature maps from the *i*-th selected layer of the pre-trained VGG-19 network, and $C_{i},H_{i},W_{i}$ represent the channel, height, and width of the feature maps, respectively.

The overall total loss function used to train our model is formulated as:

(17) $$L_{total}=L_{1}+\lambda L_{per}$$

where λ is a balancing weight set to 0.01 in our experiments.

## 4. Experiments

### 4.1. Datasets and Settings

We evaluate CSGL-Former on the RRSHID [31] and SateHaze1K [32].

**RRSHID.** RRSHID encompasses 3053 pairs of real-world hazy and haze-free RS images, allocated as 2441 for training, 304 for testing, and 308 for validation, constituting the largest real-world dataset of its kind. These pairs are stratified by haze density into three categories: thin haze (763 pairs), moderate haze (1526 pairs), and thick haze (764 pairs).

**SateHaze1K.** A synthetic dataset includes 1200 hazy and haze-free image pairs, with 960 used for training, 135 for testing, and 105 for validation. These pairs are further divided into thin haze, moderate haze, and thick haze. We trained on the entire SateHaze1K and tested on these three subsets of different concentrations.

### 4.2. Experimental Settings

Implementation Details: All models are implemented in PyTorch2.5.0 and trained on a single NVIDIA RTX 3090 Ti GPU. The architectural configuration is precisely defined as follows: the base channel dimension is set to C=48. The encoder stages progressively expand the channels to {48, 96, 192, 384}, while the decoder stages compress them to {96, 24, 6}. Across all attention modules, the number of attention heads is uniformly set to 8, with a stripe size of s=4 and a dropout rate of 0.05.

Training Details: Images are normalized to [0, 1]. We adopt the AdamW optimizer with an initial learning rate of $1\times 10^{-4}$. To stabilize the early stages of optimization, we employ a linear learning rate warmup for the first 4000 iterations. Subsequently, a Cosine Annealing decay schedule is applied, reducing the learning rate to a minimum of $1\times 10^{-6}$. The batch size is set to 2 with random 256×256 cropping. Common augmentations include random horizontal/vertical flipping and 90° rotations. The network is trained for 300 epochs. We optimize the network using the combined loss function $L_{total}=L_{1}+0.01L_{per}$.

### 4.3. Experimental Results

We conduct a comprehensive comparison of CSGL-Former against several representative methods spanning three main categories: (1) a classic prior-based method (DCP [5]); (2) advanced CNN-based models (FFA-Net [22], 4KDehazing [33]); and (3) recent Transformer-based architectures (PCSformer [25], Trinity-Net [26]). This diverse selection provides a robust benchmark across different technical paradigms. To quantitatively assess performance, we employ three widely-used full-reference image quality metrics: Peak Signal-to-Noise Ratio (PSNR), Structural Similarity Index Measure (SSIM), and Mean Squared Error (MSE). Higher PSNR and SSIM values indicate better fidelity and structural preservation, respectively, while a lower MSE reflects reduced pixel-wise reconstruction error. Furthermore, to evaluate the real-world perceptual quality, we introduce the Learned Perceptual Image Patch Similarity (LPIPS) and the Natural Image Quality Evaluator (NIQE).

Table 1 summarizes the quantitative results on the RRSHID (real-world) dataset, and Table 2 presents the results on the SateHaze1K (synthetic) dataset. On the challenging real-world RRSHID dataset, our retrained CSGL-Former achieves the highest PSNR (24.23 dB), surpassing the next-best method, Trinity-Net, by a significant margin of 1.22 dB. It also secures the best SSIM (0.6944) and the lowest MSE (0.0053). This demonstrates that our model effectively minimizes pixel-wise error while establishing superior structural preservation. On the SateHaze1K benchmark, which features dense and challenging synthetic haze, CSGL-Former maintains dominant performance, particularly achieving the highest SSIM across all three haze density levels (e.g., 0.9364 on moderate haze). It also consistently delivers excellent LPIPS and NIQE scores, highlighting the strong generalization capability of our cross-stripes global modeling and adaptive local enhancement. Following convention, the best result for each metric is marked in **bold**, and the second-best is underlined.

**Discussion on Metric Limitations and Real-World Perception.** While CSGL-Former achieves state-of-the-art PSNR and SSIM on RRSHID dataset, as well as the highest SSIM on the SateHaze1K dataset, it is crucial to discuss the inherent limitations of these metrics for practical RS analytical tasks. First, optimizing strictly for PSNR—which relies on absolute pixel-by-pixel differences—often encourages a network to output an “average” of possible high-frequency details to minimize error. This inherently leads to over-smoothed textures and blurred edges. However, practical RS analytical tasks (such as fine-grained classification, object detection, or segmentation) heavily depend on sharp structural contours, high-frequency textural details, and spectral consistency. Consequently, a model achieving the highest PSNR through aggressive smoothing might fail to preserve the discriminative semantic features required by downstream algorithms.

Furthermore, a notable numerical divergence exists in SSIM scores between synthetic and real-world datasets. While CSGL-Former achieves an excellent SSIM of 0.6944 on the real-world RRSHID dataset, this is naturally lower than the typical >0.85 scores observed on synthetic datasets like SateHaze1K. Unlike synthetic data—where hazy images are mathematically rendered from exact identical clear images (guaranteeing perfect spatial alignment)—RRSHID is a real-world paired dataset. The “ground truth” clear images and the “hazy” images were captured by sensors under fundamentally different physical conditions, including different sun angles, shadow distributions, sensor noise profiles, and slight temporal terrain changes. Because SSIM is hypersensitive to local structural misalignments, micro-contrast changes, and global illumination shifts, these inherent physical inconsistencies naturally cap the maximum achievable SSIM score. Therefore, a score of ∼0.69 on such a complex real-world dataset actually represents an exceptionally high degree of structural restoration.

To validate our model’s real-world perceptual quality beyond pixel-wise and structurally-sensitive metrics, we explicitly utilize LPIPS and NIQE. In real-world downstream RS applications, there is often no historical “ground truth” to compare against; algorithms rely purely on the geometric sharpness and spectral naturalness of the input. As shown in our quantitative results, CSGL-Former achieves highly competitive (and often the lowest) LPIPS and NIQE scores across the datasets. This explicitly proves that our method avoids over-smoothing, successfully preserves high-frequency geometric structures, and produces the most perceptually natural and feature-rich outputs, ensuring highly reliable pre-processing for real-world downstream visual analytics.

**Figure 3:**
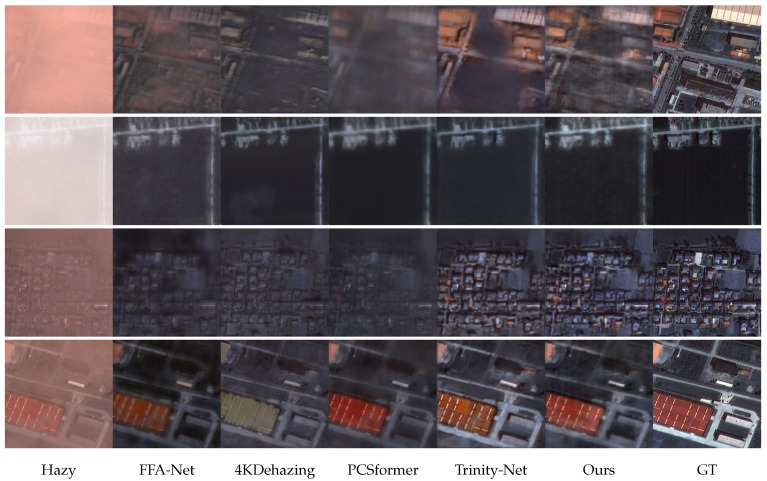
Visual samples generated by the evaluated methods on the RRSHID dataset. Bottom row lists the method order: Hazy, FFA-Net, 4KDehazing, PCSformer, Trinity-Net, Ours, and Ground Truth (GT).

**Figure 4:**
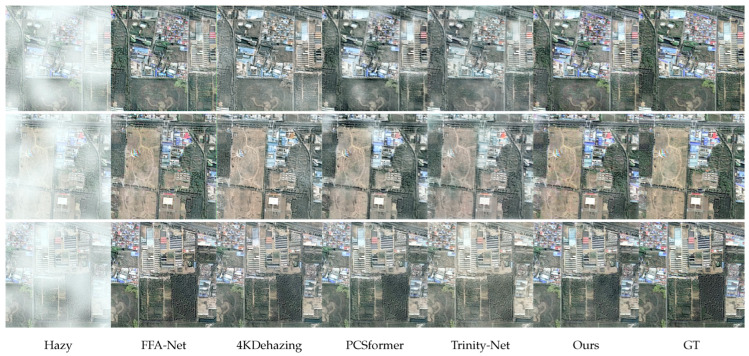
Visual samples generated by the evaluated methods on the SateHaze1K dataset. Bottom row lists the method order: Hazy, FFA-Net, 4KDehazing, PCSformer, Trinity-Net, Ours, and Ground Truth (GT).

We present side-by-side visual comparisons on the RRSHID and SateHaze1K datasets in Figure 3 and Figure 4, respectively. The columns display the original hazy input, results from representative CNN and Transformer baselines, our CSGL-Former’s output, and the ground truth. Across diverse scenes including urban blocks, vegetated regions with varying haze densities, several trends are apparent. Our method consistently recovers clearer skies and building facades without introducing color cast. Furthermore, fine structures, such as rooftop edges, are better preserved and exhibit fewer artifacts compared to the results from other methods. In scenes with heavy haze where Trinity-Net successfully maintains the main structure but tends to leave a subtle veil of haze, our method achieves a more thorough haze removal and restores global contrast. These qualitative observations perfectly align with our quantitative perceptual metrics (LPIPS/NIQE) and demonstrate our model’s superiority in preserving the radiometric and structural integrity vital for RS analysis.

### 4.4. Ablation Study

To thoroughly validate the effectiveness and necessity of the core components in our proposed CSGL-Former, we conduct comprehensive ablation studies on the real-world RRSHID dataset. We evaluate the model variants using PSNR, SSIM, and LPIPS metrics. The baseline model is constructed as a plain U-Net architecture utilizing basic cross-stripes attention and standard convolutions without our specialized modules. The quantitative results are summarized in Table 3.

**Effectiveness of the Baseline.** The basic encoder–decoder baseline achieves the lowest performance (21.42 dB PSNR and 0.6383 SSIM). This indicates that without task-specific architectural designs, a naive network struggles to handle the complex, non-homogeneous haze inherent to remote sensing imagery.

**Effectiveness of MLGA.** When the Multi-Layer Global Aggregation (MLGA) block is removed (reverting to simple sequential attention layers without dynamic weighting), the PSNR drops significantly from 24.23 dB to 23.08 dB. This substantial decline strongly supports our hypothesis: simple spatial attention is insufficient for hierarchical global reasoning. The dynamic, data-dependent weights generated by MLGA are crucial for accumulating appropriate global semantics across layers, enabling the network to effectively estimate the highly anisotropic atmospheric veil.

**Effectiveness of CGLF.** Replacing our Cross-Channel Global-Local Fusion (CGLF) module with standard feature concatenation causes the PSNR to drop to 23.15 dB, and the SSIM decreases to 0.6889. This demonstrates that naive concatenation fails to effectively bridge the semantic gap between the encoder’s global atmospheric context and the decoder’s high-frequency local details. By adaptively merging these streams with learned, data-dependent weights, CGLF ensures a more intelligent unmixing of scene radiance and haze distribution.

**Effectiveness of ALEM.** Removing the Adaptive Local Enhancement Module (ALEM) results in a noticeable performance drop (PSNR decreases to 23.20 dB). While the global stream effectively captures long-range dependencies, its large receptive field tends to over-smooth fine-grained structures. ALEM successfully counteracts this by acting as a high-frequency restorer, adaptively enhancing local geometric textures (e.g., edges and small targets) based on local haze severity.

**Effectiveness of Soft Reconstruction.** We also evaluate a variant that directly synthesizes RGB pixels instead of utilizing our Soft Reconstruction mechanism ($I_{out}=K⊙I_{in}+B+I_{in}$). Removing this mathematical constraint drops the PSNR to 23.11 dB and notably degrades the perceptual quality (LPIPS worsens to 0.4659). This degradation confirms our theoretical claim: explicitly restricting the solution space to a spatially varying affine transformation is essential. It prevents the network from hallucinating textures or over-enhancing, thereby strictly preserving the radiometric and structural integrity of the original RS image.

**Conclusion.** The complete CSGL-Former achieves the best performance across all metrics (24.23 dB PSNR, 0.6944 SSIM, and 0.4433 LPIPS). These ablation results clearly demonstrate that the proposed MLGA, ALEM, CGLF, and Soft Reconstruction modules are not merely isolated additions, but constitute a tightly coupled, synergistic system tailored for the specific physical challenges of RS image dehazing.

### 4.5. Limitations and Practical Deployment

While CSGL-Former demonstrates strong performance, we acknowledge an inherent paradigm gap between supervised restoration models trained on paired data and their deployment in real-world remote sensing (RS) scenarios, where pristine ground-truth images are strictly unavailable.

To mitigate the synthetic-to-real domain shift, our training paradigm deliberately leverages the RRSHID dataset. Unlike purely synthetic datasets (e.g., SateHaze1K), RRSHID encompasses pairs of real-world hazy and haze-free RS images that capture authentic atmospheric scattering, natural illumination variations, and complex terrain features. Training on such real-world pairs ensures that the learned mapping is much closer to actual physical degradation than models trained exclusively on synthetic physics-based rendering.

However, limitations remain in completely novel RS environments, such as newly deployed satellite sensors with different spectral responses or unseen extreme atmospheric phenomena (e.g., dense sandstorms). In these out-of-distribution, zero-shot scenarios, the absence of ground-truth clean images prevents standard fine-tuning using $L_{1}$ or $L_{2}$ losses. Consequently, the model relies entirely on its generalization capacity and may leave residual haze or introduce slight color shifts if the target haze distribution drastically differs from the training manifold.

To address how CSGL-Former can be practically deployed in real RS pipelines without ground truth, we propose two viable deployment strategies:

- Off-the-shelf Pre-processing: Thanks to the robust global–local representation learned from the large-scale real-world RRSHID dataset, CSGL-Former can act as a frozen, plug-and-play pre-processing module. It can reliably enhance the visibility of incoming hazy streams before they are fed into downstream high-level task models (e.g., land-cover classification or object detection), which are often more robust to minor residual artifacts than the human eye.
- Unsupervised Test-Time Adaptation: For sustained deployment in a specific geographic region, our architecture can serve as a strong pre-trained backbone. It can be adapted using non-reference loss functions—such as Dark Channel Prior regularization, Total Variation loss, or contrastive unpaired translation techniques—to continually align with the target domain’s statistics without requiring paired clean images.

Ultimately, to fully leverage the vast amount of unlabeled real-world hazy data available, exploring semi-supervised and self-supervised learning paradigms remains a crucial trajectory for future work, as will be further discussed in the conclusion.

## 5. Conclusions

In this paper, we introduced CSGL-Former, a novel Cross-Stripes Global–Local Fusion Transformer specifically designed for remote sensing image dehazing. Our model effectively captures anisotropic long-range dependencies by coupling cross-stripes attention with a Multi-Layer Global Aggregation strategy. It integrates a lightweight decoder featuring adaptive local enhancement and cross-channel global–local fusion to ensure the fidelity of fine-grained details. Finally, a content-preserving soft reconstruction head applies a spatially varying affine correction to remove haze while maintaining structural integrity.

Extensive experiments conducted on the real-world RRSHID and synthetic SateHaze1K datasets demonstrate the superiority of our approach. CSGL-Former achieves state-of-the-art or highly competitive performance, outperforming representative CNN and Transformer-based baselines across all standard metrics. Qualitative comparisons further corroborate these findings, showing that our method produces visually superior results with better color fidelity, enhanced detail preservation, and more thorough haze removal.

Beyond benchmark metrics, the architectural advantages of CSGL-Former—particularly its strict preservation of high-frequency geometric structures and radiometric consistency under non-homogeneous haze—translate directly into tangible benefits for real-world remote sensing workflows. As evidenced by our strong perceptual and no-reference quality evaluations (e.g., LPIPS and NIQE), which serve as robust proxy indicators for downstream success, our method significantly improves the operational reliability of downstream applications by acting as a high-fidelity pre-processing stage. This includes time-critical scenarios like disaster response, where clear aerial visibility is paramount, as well as broader tasks such as environmental monitoring and intelligent transportation systems relying on UAVs or satellite imagery.

While comprehensively integrating and fine-tuning standard downstream validation pipelines remains beyond the immediate scope of this physical restoration study, addressing this application gap is our primary trajectory for future research. Future work will proceed in several promising directions: (i) building a unified “Dehazing-for-Detection” framework to explicitly validate the performance of CSGL-Former on standard RS classification and object detection benchmarks (e.g., DOTA and FAIR1M); (ii) extending the architecture to handle multi-spectral and hyperspectral RS imagery; (iii) enhancing the model’s robustness to extreme radiometric conditions and illumination variations, such as sun glare; and (iv) exploring semi- and self-supervised learning paradigms to better leverage the vast amount of unlabeled real-world hazy data available. 

## Figures and Tables

**Table 1:** Quantitative comparison on RRSHID. Best and second best are in **bold** and underlined, respectively. ↑ indicates higher is better, and ↓ indicates lower is better.

Method	RRSHID-Average				
	PSNR (↑)	SSIM (↑)	MSE (↓)	LPIPS (↓)	NIQE (↓)
DCP	13.37	0.4200	0.0629	0.5189	**6.3804**
FFA-Net	22.10	0.6481	0.0092	0.4458	7.8363
4KDehazing	22.79	0.6738	0.0073	0.4595	9.5837
PCSformer	22.93	0.6431	0.0073	0.4811	16.4746
Trinity-Net	23.01	0.6772	0.0075	**0.4438**	8.9529
**CSGL-Former (Ours)**	**24.23**	**0.6944**	**0.0053**	0.4444	9.1539

**Table 2:** Quantitative comparison on SateHaze1K. Best and second best are in **bold** and underlined, respectively. ↑ indicates higher is better, and ↓ indicates lower is better.

Method	SateHaze1K-Thin					SateHaze1K-Moderate					SateHaze1K-Thick				
	PSNR (↑)	SSIM (↑)	MSE (↓)	LPIPS (↓)	NIQE (↓)	PSNR (↑)	SSIM (↑)	MSE (↓)	LPIPS (↓)	NIQE (↓)	PSNR (↑)	SSIM (↑)	MSE (↓)	LPIPS (↓)	NIQE (↓)
DCP	20.55	0.8749	0.0091	0.0703	3.9555	21.39	0.9147	0.0083	0.0662	4.0169	16.47	0.7575	0.0238	0.1552	3.639
FFA-Net	23.53	0.9077	0.0046	0.0665	4.2221	24.64	0.9327	0.0036	0.0569	4.7465	**21.91**	0.8399	**0.0066**	0.1331	4.4210
4KDehazing	22.20	0.9042	0.0066	0.0597	4.5268	**26.33**	0.9305	**0.0026**	0.0570	4.6216	20.41	0.8139	0.0093	0.1665	4.1614
PCSformer	22.50	0.8927	0.0057	0.0623	3.9616	24.95	0.9301	0.0034	0.0540	4.0704	20.68	0.8108	0.0086	0.1322	3.4181
Trinity-Net	**23.58**	0.9016	**0.0044**	0.0706	4.0962	23.81	0.9191	0.0045	0.0820	4.2092	16.37	0.7694	0.0233	0.1888	3.8917
**CSGL-Former (Ours)**	23.34	**0.9088**	0.0047	**0.0583**	**3.8126**	25.32	**0.9364**	0.0033	**0.0525**	**3.9392**	20.82	**0.8416**	0.0084	**0.1308**	**3.3407**

**Table 3:** Ablation study of different components on RRSHID dataset. Best and second best are in **bold** and underlined, respectively. ↑ indicates higher is better, and ↓ indicates lower is better.

Method	RRSHID-Average		
	PSNR (↑)	SSIM (↑)	LPIPS (↓)
CSGLFormer	**24.23**	**0.6944**	**0.4433**
w/o ALEM	23.20	0.6834	0.4487
w/o CGLF	23.15	0.6889	0.4537
w/o MLGA	23.08	0.6824	0.4524
w/o soft reconstruction	23.11	0.6841	0.4659
baseline	21.42	0.6383	0.4900

## Data Availability

The RRSHID and SateHaze1K datasets used in this study are publicly available resources. The original data of RRSHID are openly available in GithUb at https://github.com/AeroVILab-AHU/RRSHID (accessed on 15 October 2025). The original data of SateHaze1K are openly available in Kaggle at https://www.kaggle.com/datasets/xuxingxing233/satehaze1k (accessed on 15 October 2025).
